# A theory-based assessment of mpox: Findings from a nationally representative survey of U.S. adults

**DOI:** 10.1371/journal.pone.0299599

**Published:** 2024-03-15

**Authors:** Margaret L. Walsh-Buhi, Rebecca F. Houghton, Danny Valdez, Eric R. Walsh-Buhi

**Affiliations:** 1 Center for Collaborative Systems Change, Indiana University-Bloomington, Bloomington, Indiana, United States of America; 2 Department of Applied Health Science, Indiana University School of Public Health-Bloomington, Bloomington, Indiana, United States of America; Instituto Nacional de Perinatologia, MEXICO

## Abstract

The purpose of this research was to examine individual differences related to fear of, perceived susceptibility to, and perceived severity of mpox as well as mpox knowledge, fear, perceived susceptibility, and perceived severity as predictors of vaccine intention in a national survey of U.S. adults (aged ≥18 years). Address-based sampling (ABS) methods were used to ensure full coverage of all households in the nation, reflecting the 2021 March Supplement of the Current Population Survey. Internet-based surveys were self-administered by Ipsos between September 16–26, 2022. N = 1018 participants completed the survey. The survey included items, based partially on the Health Belief Model, assessing vaccine intention (1 item; responses from 1 [Definitely not] to 5 [Definitely]), fear of mpox (7-item scale; α = .89; theoretical mean = 7–35), perceived susceptibility to mpox (3-item scale; α = .85; theoretical mean = 3–15), and perceived severity of mpox (4-item scale; α = .65; theoretical mean = 4–20). Higher scores indicate greater fear, susceptibility, and severity. One-way ANOVAs were run to examine mean score differences by demographic groups (e.g., gender, race/ethnicity, sexual orientation), and multiple regression analyses assessed the relationship between predictors (mpox knowledge, susceptibility/severity, fear) and a single outcome (vaccination intention), while controlling for demographic covariates. Sampling weights were applied to all analyses. Only 1.8% (n = 18) of respondents reported having received the mpox vaccine. While mpox vaccine intention was low (M = 2.09, SD = 0.99), overall differences between racial/ethnic, sexual orientation, education, and household income groups were statistically significant. Fear of mpox was very low (M = 13.13, SD = 5.33), and there were overall statistically significant differences in both fear and perceived severity among gender, race/ethnicity, sexual orientation, education, and household income groups. While respondents reported not feeling very susceptible to mpox (M = 5.77, SD = 2.50), they generally rated mpox as just above the theoretical mean in terms of severity (M = 11.01, SD = 2.85). Mpox knowledge, fear, severity, and susceptibility, as well as race/ethnicity, were all statistically significant predictors of intention to vaccinate, with susceptibility representing the strongest predictor. Overall, Americans’ vaccination for mpox/vaccine intent was low. Gay/lesbian and racial/ethnic minority respondents felt more susceptible to and viewed mpox more severely, compared with heterosexual and White respondents, respectively. These data may be used to tailor risk and prevention (e.g., vaccination) interventions, as cases continue to surge in the current global mpox outbreak. Greater perceptions of susceptibility, severity, and fear about mpox exist largely among minority populations. While public health messaging to promote mpox vaccination can focus on improving knowledge, as well as addressing fear and perceived severity of, and susceptibility to, mpox, such messages should be carefully crafted to prevent disproportionate negative effects on marginalized communities.

## Introduction

Human monkeypox (mpox) [1] is a disease caused by infection with the monkeypox virus, part of the same family of viruses as variola virus—the virus that causes smallpox [2]. Prior to April 2022, mpox had been endemic in several central and western African countries [3,4]. Previously, almost all mpox cases in people outside of Africa were linked to international travel to countries where the disease commonly occurs or through animals imported from those countries [2].

The most recent mpox outbreak began April 27, 2022 in the United Kingdom [4]. In the U.S., the first mpox case of the current outbreak was identified on May 18, 2022 [3]. Worldwide, 528 mpox infections were diagnosed between April 27 and June 24, 2022, in 16 countries and, overall, 98% of the persons with infection were gay or bisexual men [5]. As of July 2023, more than 95% of cases have been among men [6] and primarily men-who-have-sex-with-men [7]. As of January 11, 2024, there have been 31,689 cases reported in the U.S. and 88,026 globally [6]. New cases continue to occur with 124 new cases reported in the U.S. from October 23-November 23, 2023 [6]. Among the U.S. cases with available data, 41% were among non-Hispanic White (White) persons, 28% were among Hispanic or Latino (Hispanic) persons, and 26% were among non-Hispanic Black or African American persons [3]. The World Health Organization (WHO) declared mpox a public health emergency of international concern on July 24, 2022 and the U.S. declared mpox a public health emergency on August 4, 2022, freeing up resources and funding to combat the virus, including expanding vaccine promotion and raising public awareness [8,9].

Previous public health messaging around knowledge, awareness, prevention, and treatment of mpox prior to the 2022 outbreak in the U.S. has been limited. Public health officials have known that there are continued challenges to preventing reemergence and/or spread of mpox; primarily, a lack of knowledge around the disease [10]. In a recent national survey of U.S. adults [11], overall mpox knowledge was very low; respondents averaged only 41% correct on a scale of mpox knowledge. Furthermore, differences in knowledge by sexual orientation, urbanicity, education, and household income groups were statistically significant. Mpox knowledge was lowest among heterosexually identified, rural, and low education/income respondents [11].

Guided by the Health Belief Model (HBM) [12] and Theory of Planned Behavior (TPB) [13], in this research we focus on key theoretical constructs and concepts such as mpox knowledge, perceived susceptibility, severity, and fear, as well as other factors that may influence individual behavioral intentions and help inform mitigation strategies for mpox prevention and control. Findings from this study will underscore the potential impact of perceptions and knowledge about mpox on perceived susceptibility, severity, and fear of contracting the disease. As mpox represents another recent example of a public health emergency related to a relatively unknown or misunderstood disease, and the first following the COVID-19 pandemic, our findings draw further implications on the importance of disease-related knowledge and the need for related education. This knowledge is crucial in promoting and adhering to informed mitigation strategies, particularly in the context of COVID-19 fatigue and increased levels of public health mistrust from the general population.

## Materials and methods

The purpose of this research was to examine individual differences related to fear of, perceived susceptibility to, and perceived severity of mpox as well as mpox knowledge, fear, perceived susceptibility, and perceived severity as predictors of vaccine intention in a national survey of U.S. adults (aged ≥18 years).

### Setting and study population

This research employed the KnowledgePanel by Ipsos. KnowledgePanel is the first and largest online research panel that is representative of the entire U.S. population [14]. Ipsos recruits panel members using address-based sampling (ABS) methods to ensure full coverage of all households in the nation [15]. The mpox survey was self-administered and accessible any time of day between September 16 and September 26, 2022. N = 1018 members completed the survey, representing a 55% completion rate. Participation in the survey was completely anonymous [14]. Once all survey data were collected and processed, design weights were adjusted to account for any differential nonresponse that may have occurred.

All study procedures and data collection instruments were reviewed and approved by the lead author’s human subjects review board (IRB Protocol #16444). All KnowledgePanel members were provided a link and must have agreed to the informed consent (privacy terms) electronically before beginning the initial demographic survey. As this research involved interactions such as educational tests, surveys, interviews, or observation of public behavior (and would not reasonably place subjects at risk), the study was considered Exempt (Category 2[ii]) by the IRB. No minors (i.e., those aged <18 years) were involved in this research.

### Variables

Variables were based on existing literature, study goals, and guided by constructs from the HBM [12] and TPB [13]. At the time of survey administration, we used the term *monkeypox*. Moving forward however, mpox is now the preferred term [1] and will be used throughout this paper. For additional details on all survey items described below, refer to the Supporting Information document titled S1 File.

### Mpox knowledge

Individual differences related to mpox knowledge is reported elsewhere [11]. Mpox knowledge was assessed using a 15-item scale, with items adapted from Sallam et al [16] and Harapan et al [17]. Correct responses were scored as 1, incorrect responses were scored as −1, and “I do not know” was given a score of zero, which was used as a sum to represent the mpox knowledge score (with a higher score indicating greater knowledge of mpox).

### Perceived susceptibility and perceived severity

Perceived susceptibility and perceived severity were assessed using 3- and 4-item scales (ranging from 1 [“Strongly disagree”] to 5 [“Strongly agree”]), respectively, adapted from Niculaescu et al [18]. Perceived susceptibility sample items included “I am at risk of getting mpox” and “It is likely that I will get mpox” (α = .85). The perceived susceptibility scale theoretical range was between 3 and 15, with a higher score indicating a greater susceptibility to mpox. Perceived severity sample items included “If I get mpox, I will get sick” and “If I get mpox, I will die” (α = .65). The perceived severity scale theoretical range was between 4 and 20, with a higher score indicating that mpox is viewed as more severe.

### Fear of mpox

Fear of mpox was assessed using a 7-item scale, adapted from the *Fear of COVID-19 Scale*, developed by Ahorsu et al [19]. Sample items included “I am afraid of mpox” and “When seeing news stories about mpox, I become nervous or anxious.” Responses were based on a 7-point scale, ranging from “Strongly disagree” to “Strongly agree” (the fear of mpox scale theoretical range was between 7 and 49, with a higher score indicating a greater fear of mpox). Score reliability for these data was α = .89.

### Vaccination and vaccine intention

Mpox and smallpox vaccination history was assessed with 2 items (no/yes/not sure) and mpox vaccine intention was assessed with a single item, with response options ranging from “Definitely not” to “Definitely” (ranging from 1 to 5, with a higher score indicating a stronger intent).

### Demographics

The KnowledgePanel included a number of demographic variables, including gender, race/ethnicity, sexual orientation, metropolitan statistical area (or MSA) status, Census 4 Region, education, and household income. For additional details on these socio-demographic, educational, and ethnicity-related items, refer to S2 File.

### Statistical analyses

Basic frequencies and descriptive statistics (e.g., means, standard deviations [SDs]) were conducted on all variables using IBM SPSS Statistics 28. Tests of normal distribution (i.e., skewness and kurtosis) and other tests were conducted on all relevant variables to assess whether assumptions were met for subsequent correlational analyses. All data were approximately normally distributed. One-way ANOVAs were run to examine mean score differences in mpox fear, perceived susceptibility, perceived severity, and vaccine intention by the following demographic groups: gender, race/ethnicity, sexual orientation, MSA status, Census Region, education, and household income. As statistically significant differences were found, multiple comparisons were then conducted to determine between which demographic categories differences existed in the data (e.g., for race/ethnicity: White, Non-Hispanic compared with Black or African American, Non-Hispanic). Lastly, we performed a multiple regression to assess the relationship between predictors (mpox knowledge, susceptibility/severity, and fear) and a single outcome (vaccination intention), while controlling for covariates (sexual orientation, gender, education, and race/ethnicity). Sampling weights were applied to all analyses.

## Results

Weighted and unweighted sociodemographic characteristics of the sample are displayed in Table 1. Table 2 displays the correlation coefficients for knowledge, susceptibility, severity, and fear of mpox. Responses to the fear of mpox, perceived susceptibility to mpox, and perceived severity of mpox scales are displayed in Table 3. Regression results are displayed in Table 4. ANOVAs of perceived susceptibility of and perceived severity to mpox, fear of mpox, and vaccine intention across demographic variables can be found in Table 5.

**Table 1:** Participants’ sociodemographic characteristics, U.S., September 2022 (N = 1018, unless otherwise noted).

Gender	N	Unweighted %	Weighted %
Male	516	50.7	48.5
Female	502	49.3	51.5
Race/Ethnicity			
White, Non-Hispanic	708	69.5	62.5
Black or African American, Non-Hispanic	98	9.6	12.0
Other, Non-Hispanic	50	4.9	4.8
Hispanic	121	11.9	16.9
2+ races, Non-Hispanic	41	4.0	3.8
Education			
No high school diploma or GED	76	7.5	9.3
High school graduate (high school diploma or the equivalent GED)	291	28.6	28.6
Some college or Associate degree	276	27.1	27.1
Bachelor’s degree	209	20.5	20.6
Master’s degree or above	166	16.3	14.3
Household Income			
Under $10,000	35	3.4	5.1
$10,000 to $24,999	62	6.1	7.7
$25,000 to $49,999	157	15.4	17.0
$50,000 to $74,999	165	16.2	16.3
$75,000 to $99,999	150	14.7	13.2
$100,000 to $149,999	203	19.9	17.9
$150,000 or more	246	24.2	22.8
Marital Status			
Now married	594	58.3	52.4
Widowed	58	5.7	4.7
Divorced	98	9.6	9.4
Separated	14	1.4	1.6
Never married	254	25.0	32.0
Sexual Orientation (N = 952)			
Gay or lesbian	40	4.2	4.3
Straight, that is, not gay	876	92.0	90.5
Bisexual	21	2.2	2.8
Something else	15	1.6	2.3
Metropolitan Statistical Area (MSA)			
Non-metro area	135	13.3	13.0
Metro area	883	86.7	87.0
U.S. Census Region			
Northeast	170	16.7	17.2
Midwest	219	21.5	20.6
South	379	37.2	38.3
West	250	24.6	23.9

**Table 2:** Summary statistics for variables analyzed and bivariate correlations: U.S., September 2022.

	N	Mean (SD)	Knowledge	Fear (95% CI)	Perceived Susceptibility (95% CI)	Perceived Severity (95% CI)
Knowledge	1012	5.71 (3.85)	1	-.055 (-.117, .006; N = 1006)	-.103 (-.164, -.041; N = 1006)	.132 (.071, .192; N = 1006)
Fear	1008	13.13 (5.33)	--	1	.630 (.591, .665; N = 1008)	.586 (.544, .626; N = 1008)
Perceived Susceptibility	1008	5.77 (2.50)	--	--	1	.489 (.441, .535; N = 1008)
Perceived Severity	1008	11.01 (2.85)	--	--	--	1

*Note*. CI  =  confidence interval. Any confidence interval that is inclusive of zero is not statistically significant at p = .05. Correlations are Pearson Correlations.

**Table 3:** Perceived susceptibility to monkeypox, perceived severity of monkeypox, and fear of monkeypox item means and Standard Deviations (SDs).

*Perceived Susceptibility to Monkeypox* (α = .85)		
I am at risk of getting monkeypox.	998	2.00 (0.979)
It is likely that I will get monkeypox.	1001	1.82 (0.845)
Individuals in my household are at risk of getting monkeypox.	1000	2.00 (1.015)
*Perceived Severity of Monkeypox* (α = .65)		
I believe that monkeypox is a severe health problem.	1003	2.94 (1.054)
If I get monkeypox, I will get sick.	995	3.32 (0.972)
If I get monkeypox, I will die.	997	1.97 (0.904)
If I get monkeypox, other members in my household will get sick.	1001	2.87 (1.036)
*Fear of Monkeypox* (α = .89)	N	Mean (SD)
I am afraid of monkeypox.	1001	2.26 (1.127)
It makes me uncomfortable to think about monkeypox.	1001	2.29 (1.146)
My hands become clammy when I think about monkeypox.	1003	1.67 (0.858)
I am afraid of losing my life because of monkeypox.	997	1.79 (0.946)
When seeing news stories about monkeypox, I become nervous or anxious.	1004	2.05 (1.040)
I cannot sleep because I’m worrying about getting monkeypox.	1001	1.46 (0.745)
My heart races or palpitates when I think about getting monkeypox.	1002	1.71 (0.911)

*Note*. Theoretical range = 1 to 5, with 5 indicating stronger agreement with the item/statement (i.e., greater fear of monkeypox, greater perceived susceptibility, or greater perceived severity).

**Table 4:** Multiple regression models predicting mpox vaccination intentions.

**Model 1: Controls Only**	**B**		**SE**		**β**		**T**	***p***	**95% CI**	**R** ^**2**^
Education	-0.02		0.03		-0.02		-0.56	0.57	-.08, .04	
Race/Ethnicity	0.19		0.03		0.25		7.43	< .001	.14, .23	
Gender	0.11		0.07		0.05		1.57	0.116	-.03, .24	
Household Income	-0.03		0.02		-0.06		-1.60	0.109	-.07, .01	
Sexual Orientation	-0.13		0.08		-0.05		-1.57	0.117	-.29, .03	
Constant	2.09		0.23				9.15	< .001	1.64, 2.53	
										**0.07**
**Model 2: Controls and Predictors**	**B**		**SE**		**β**		**T**	***p***		**R** ^**2**^
Mpox Knowledge	0.03		0.01		.114		3.59	< .001	.01, .05	
Mpox Fear	0.02		0.01		0.13		3.00	0.003	.01, .04	
Mpox Severity	0.03		0.01		0.09		2.19	0.03	.003, .06	
Mpox Susceptibility	0.09		0.02		0.23		5.95	< .001	.06, .12	
Education	0.004		0.03		0.005		0.15	0.88	-.05, .06	
Race/Ethnicity	0.14		0.02		0.19		5.97	< .001	.09, .18	
Gender	0.01		0.06		0.006		0.20	0.84	-.11, .13	
Household Income	-0.02		0.02		-.028		-0.83	0.41	-.05, .02	
Sexual Orientation	-0.12		0.08		-.048		-1.56	0.12	-.27, .03	
Constant	0.78		0.24				3.30	.001	.32, 1.24	
										**0.23**

**Table 5:** ANOVAs of perceived susceptibility to mpox, and perceived severity of mpox, fear of mpox, and vaccine intention across demographic variables.

			Sum of Squares	df	Mean Square	F	Significance
Perceived susceptibility	Gender	Between groups	12.666	1	12.666	2.035	.154
		Within groups	6254.446	1005	6.223		
		Total	6267.112	1006			
	Race/ethnicity	Between groups	145.597	4	36.399	5.958	< .001
		Within groups	6121.515	1002	6.109		
		Total	6267.112	1006			
	Sexual orientation	Between groups	155.235	3	51.745	8.386	< .001
		Within groups	5756.901	933	6.170		
		Total	5912.136	936			
	MSA status	Between groups	11.845	1	11.845	1.903	.168
		Within groups	6255.267	1005	6.224		
		Total	6267.112	1006			
	Census Region	Between groups	4.641	3	1.547	.248	.863
		Within groups	6262.470	1003	6.244		
		Total	6267.112	1006			
	Education	Between groups	177.635	4	44.409	7.307	< .001
		Within groups	6089.477	1002	6.077		
		Total	6267.112	1006			
	Household income	Between groups	169.510	6	28.252	4.633	< .001
		Within groups	6097.602	1000	6.098		
		Total	6267.112	1006			
Perceived Severity	Gender	Between groups	171.416	1	171.416	21.595	< .001
		Within groups	7977.539	1005	7.938		
		Total	8148.954	1006			
	Race/ethnicity	Between groups	122.261	4	30.565	3.816	.004
		Within groups	8026.693	1002	8.011		
		Total	8148.954	1006			
	Sexual orientation	Between groups	178.228	3	59.409	7.484	< .001
		Within groups	7406.094	933	7.938		
		Total	7584.322	936			
	MSA status	Between groups	1.569	1	1.569	.194	.660
		Within groups	8147.385	1005	8.107		
		Total	8148.954	1006			
	Census Region	Between groups	9.680	3	3.227	.398	.755
		Within groups	8139.274	1003	8.115		
		Total	8148.954	1006			
	Education	Between groups	102.339	4	25.585	3.186	.013
		Within groups	8046.615	1002	8.031		
		Total	8148.954	1006			
	Household income	Between groups	175.992	6	29.332	3.679	.001
		Within groups	7972.962	1000	7.973		
		Total	8148.954	1006			
Fear	Gender	Between groups	577.615	1	577.615	20.699	< .001
		Within groups	28073.473	1006	27.906		
		Total	28651.088	1007			
	Race/ethnicity	Between groups	2734.390	4	683.597	26.456	< .001
		Within groups	25916.698	1003	25.839		
		Total	28651.088	1007			
	Sexual orientation	Between groups	584.412	3	194.804	6.967	< .001
		Within groups	26117.368	934	27.963		
		Total	26701.780	937			
	MSA status	Between groups	.650	1	.650	.023	.880
		Within groups	28650.438	1006	28.480		
		Total	28651.088	1007			
	Census Region	Between groups	187.906	3	62.635	2.209	.085
		Within groups	28463.181	1004	28.350		
		Total	28651.088	1007			
	Education	Between groups	1562.744	4	390.686	14.466	< .001
		Within groups	27088.344	1003	27.007		
		Total	28651.088	1007			
	Household income	Between groups	1874.313	6	312.385	11.678	< .001
		Within groups	26776.775	1001	26.750		
		Total	28651.088	1007			
Mpox vaccine intention	Gender	Between groups	1.915	1	1.915	1.959	.162
		Within groups	892.289	913	.977		
		Total	894.204	914			
	Race/ethnicity	Between groups	63.858	4	15.964	17.496	< .001
		Within groups	830.346	910	.912		
		Total	894.204	914			
	Sexual orientation	Between groups	16.366	3	5.455	5.887	< .001
		Within groups	785.741	848	.927		
		Total	802.107	851			
	MSA status	Between groups	1.835	1	1.835	1.877	.171
		Within groups	892.369	913	.977		
		Total	894.204	914			
	Census Region	Between groups	1.303	3	.434	.443	.722
		Within groups	892.901	911	.980		
		Total	894.204	914			
	Education	Between groups	23.113	4	5.778	6.036	< .001
		Within groups	871.091	910	.957		
		Total	894.204	914			
	Household income	Between groups	23.069	6	3.845	4.007	< .001
		Within groups	871.135	908	.959		
		Total	894.204	914			

### Perceived susceptibility

Perceived susceptibility to mpox was very low (M = 5.77, SD = 2.50; Table 3). There were statistically significant overall differences in perceived susceptibility to mpox by race/ethnicity, sexual orientation, education, and household income groups (Table 5).

### Multiple comparisons: Perceived susceptibility

When compared with their White (non-Hispanic) counterparts (M = 5.5, SD = 2.43), Black or African American (non-Hispanic) (M = 6.5, SD = 2.51) and Hispanic (M = 6.2, SD = 2.64) respondents perceived themselves to be more susceptible to mpox. Gay or lesbian respondents (M = 7.2, SD = 2.48) reported feeling more susceptible, compared with their straight/heterosexual counterparts (M = 5.7, SD = 2.49). Respondents with no high school diploma or GED (M = 6.8, SD = 2.63) reported feeling more susceptible, compared with all other education groups. High school graduates (M = 6.0, SD = 2.55) reported feeling more susceptible, compared with respondents with a Master’s degree or above (M = 5.1, SD = 2.08). Respondents reporting household incomes under $10,000 reported feeling more susceptible, compared with all income groups making ≥$25,000. No statistically significant differences in perceived susceptibility were found by gender, MSA status, or Census Region groups.

### Perceived severity

While respondents reported not feeling very susceptible to mpox, they generally rated mpox as just above the theoretical mean in terms of severity (M = 11.01, SD = 2.85; Table 3). There were statistically significant overall differences in perceived severity of mpox by gender, race/ethnicity, sexual orientation, education, and household income groups (Table 5).

### Multiple comparisons: Perceived severity

When compared with their male counterparts (M = 10.60, SD = 2.95), females (M = 11.4, SD = 2.69) perceived mpox more severely. Hispanic (M = 11.4, SD = 3.23) respondents perceived mpox more severely than their White (non-Hispanic) counterparts (M = 10.8, SD = 2.70). Gay or lesbian (M = 12.1, SD = 2.32) and bisexual (M = 12.9, SD = 2.06) respondents perceived mpox more severely, compared with their straight/heterosexual counterparts (M = 10.9, SD = 2.85). Respondents with no high school diploma or GED (M = 12.0, SD = 3.00) perceived mpox more severely than did high school graduates (M = 10.8, SD = 2.94), respondents with some college or an Associate’s degree (M = 10.9, SD = 2.78), and those with a Bachelor’s degree (M = 11.0, SD = 2.87). Respondents reporting household incomes under $10,000 perceived mpox more severely, compared with income groups making ≥$100,000. No statistically significant differences in perceived severity were found by MSA status or Census Region groups.

### Fear of mpox

Fear of mpox was very low (M = 13.13, SD = 5.33; Table 3). There were statistically significant overall differences in mpox fear by gender, race/ethnicity, sexual orientation, education, and household income groups (Table 5).

### Multiple comparisons: Fear of mpox

When compared with their male counterparts (M = 12.4, SD = 5.22), females (M = 13.9, SD = 5.34) were more fearful of mpox. Black or African American (non-Hispanic) (M = 16.1, SD = 5.23), Other (non-Hispanic) (M = 15.5, SD = 5.28), and Hispanic (M = 14.5, SD = 5.85) respondents were more fearful, compared with their White (non-Hispanic) counterparts (M = 11.9, SD = 4.79). When compared with straight/heterosexual respondents (M = 12.9, SD = 5.23), those identifying as “something else” (M = 17.7, SD = 5.72) were also more fearful.

Respondents with no high school diploma or GED (M = 16.1, SD = 5.83) were statistically more fearful of mpox, compared with all other education groups. High school graduates (M = 13.8, SD = 5.62) were more fearful, compared with respondents with a Bachelor’s degree (M = 12.4, SD = 5.37) or a Master’s degree or above (M = 11.4, SD = 4.1). Those with some college or an Associate’s degree (M = 12.9, SD = 4.91) were more fearful, compared with respondents with a Master’s degree or above\Respondents reporting household incomes under $10,000 were more fearful, compared with all income groups making ≥$25,000. Those reporting a household income of $10,000 to $24,999 were statistically more fearful, compared with income groups making ≥$75,000, and those reporting incomes of $25,000 to $49,999 were more fearful, compared with income groups making ≥$100,000. No statistically significant differences in fear were found by MSA status or Census Region groups.

### Vaccination status and vaccine intention

Just over half of respondents (n = 506, 50.2%) reported that they have received the smallpox vaccination. Only 1.8% (n = 18) reported that they have received the mpox vaccine. Intention to get the mpox vaccine (M = 2.09, SD = 0.99) in the next 6 months was low. There were statistically significant overall differences between racial/ethnic, sexual orientation, education, and household income groups on intent to vaccinate for mpox (Table 5).

### Multiple comparisons: Vaccine intention

When compared with White (non-Hispanic) respondents (M = 1.9, SD = 0.87), Black or African American (non-Hispanic) (M = 2.3, SD = 1.13), Other (non-Hispanic) (M = 2.5, SD = 0.97), and Hispanic respondents (M = 2.5, SD = 1.13) had statistically higher scores on intention to vaccinate for mpox. When compared with straight/heterosexual respondents (M = 2.06, SD = 0.96), gay or lesbian (M = 2.68, SD = 1.04) respondents, not including bisexual respondents, had statistically higher scores on intention to vaccinate.

When compared with those respondents with more education (e.g., High school graduate, some college), respondents with No high school diploma or GED had statistically higher scores on intention to vaccinate for mpox. Respondents with a household income of less than $10,000 (M = 2.73, SD = 1.29) had statistically higher scores on intention to vaccinate, when compared with those higher income categories, such as the $50,000-$74,999 range (M = 2.11, SD = .93), $100,000-$149,999 range (M = 1.9, SD = .92), and $150,000 or more (M = 2.13, SD = .97). Similarly, those earning between $25,000 and $49,999 (M = 2.15, SD = 1.05) had higher vaccine intention scores than those in the $100,000-$149,999 range. No statistically significant differences in vaccine intention were found by gender, MSA status, or Census Region groups.

### Multiple regression: Predicting vaccine intention

Our regression analysis examined the predictors of intention to vaccinate for mpox while controlling for sexual orientation, gender, education, and race/ethnicity (Table 4). The analysis revealed that the control variables collectively had a statistically significant effect on intention to vaccinate, F(5, 844) = 13.41, p < .001. However, among the control variables, only race/ethnicity (B = .19, SE = .03, β = .25, t = 7.43, p < .001) showed a statistically significant relationship with intention to vaccinate. The control variables accounted for 7% of the total variance. Our larger regression model, which included the control variables as well as mpox knowledge, fear, severity, and susceptibility, was also statistically significant, F(5, 844) = 27.92, p < .001. Mpox knowledge, fear, severity, and susceptibility were all statistically significant predictors of intention to vaccinate, with susceptibility representing the strongest predictor (Table 4). This larger model accounted for 23% of the variance, explaining an additional 16% of the variance compared to the control-only model.

## Discussion

Across the sample, perceived susceptibility was low overall, though differences among certain demographic groups were evident: Of those respondents who reported being Black, Hispanic, gay/lesbian, or those with a high school degree (compared to those with a Master’s or above), or those making less than $10,000 (compared with all other income groups) all felt more susceptible to mpox. Severity of mpox was rated more highly by females, Hispanic individuals, gay or lesbian and bisexual individuals, those in the lowest income category, and of those with no high school degree or GED. Similarly, mpox fear was quite low across the sample, though with some statistically higher levels of fear reported among females, non-White individuals, those with lower levels of educational attainment, lower income levels, and those identifying their sexual orientation as “something else.”

In terms of vaccine uptake, only 1.8% of the sample reported having received the mpox vaccine. Intentions to vaccinate were generally low across the sample. While research indicates that individuals with higher household incomes and higher educational attainment know more about mpox (compared with lower income/education individuals),^11^ these groups scored lower on intentions to vaccinate. In this work, those in the lowest household income bracket, and those with no high school diploma or GED had *higher* intentions to vaccinate. This unexpected result may be explained in part by the Dunning-Kruger effect, a cognitive bias whereby individuals with low ability, expertise, or experience regarding a certain type of area of knowledge tend to overestimate their ability or knowledge [20]. Overall, intentions to vaccinate were higher among nonwhite respondents and those who identify as gay or lesbian.

Research indicates that around half of men-who-have-sex-with-men (MSM) [21] surveyed have curbed risky sexual activity due to mpox concerns. Yet, the risk of contracting mpox is not limited to this population; concerns over the proliferation of stigma around mpox and the gay, bisexual, and other MSM community requires development of programs that are sensitive to the prevention of stigma while tailored harm reduction practices must be pursued [3]. Focus on Given this context, it is not surprising that sexual orientation is the one demographic category where statistical differences were found across all constructs.

Results of our regression models point to valuable mpox intervention and vaccine promotion targets. For example, messaging can focus on improving mpox knowledge, as well as addressing fear and perceived severity of, and susceptibility to, mpox. Along with these findings, we also suggest that public health and health communication efforts pay close attention to racial and ethnic identities included in mpox prevention strategies.

### Limitations

There are potential limitations of this research. First, the study may have been impacted by self-report bias. Second, as this was a one-time survey in September 2022, we are uncertain as to potential impacts of a history effect on respondents’ responses. For example, due to the cross-sectional nature of the study, respondents may have been influenced *before* survey participation, either by media blitzes or federal, state, or local initiatives aimed at increasing mpox awareness and knowledge. Third, the sample, while nationally representative, may not be generalizable to certain priority populations, such as the unhoused and those who are incarcerated. Nonetheless, these findings represent the first of its kind in the U.S. regarding perceived susceptibility to mpox, perceived severity of mpox, mpox fear, and vaccine history/intention. Its reliance on behavioral theory is also a strength.

### Public health implications

National messaging regarding mpox has shifted from crisis mode to developing an ongoing, adaptable, long-term approach [22]. Though rates of mpox infection have dropped since June 2022, new mutations continue to occur [23]. The Director of the Cornell Center for Pandemic Prevention and Response recently noted, “The most likely scenario is that we will continue to see kind of a low level of [mpox] cases kind of continuously, as we do with other sexually transmitted infections and that unfortunately, it will concentrate predominantly in urban areas among young Black and brown men, and probably in various parts of the Midwest and South, similar to what we see for syphilis and HIV”[22]. Data from this study corroborate that sentiment in that greater perceptions of susceptibility, severity, and fear about mpox exist largely among such minority populations [24]. Moreover, efforts to increase sexual health literacy, address stigma, and ensure the equitable dissemination of mpox messaging and subsequent public health interventions for these population is necessary [24].

Further, newsroom skepticism when reporting on mpox minimizes potential impact [25] and a general lack of coverage on mpox is worrisome. While mpox may not threaten mortality rates in the general population to the extent that COVID-19 has in recent years, Americans must not become desensitized to the risks of mpox in the same way that we have seen Americans become fatigued by COVID-19 messaging [26,27]. As mpox becomes endemic to the U.S., public health messaging must be carefully crafted to prevent disproportionate negative effects on marginalized communities [28]. We must also be cognizant of the need to evolve prevention tactics as mpox continues to mutate and to prevent complacency as mpox cases level off, which may lead to increases in risky behaviors and a subsequent mpox resurgence [29].

## Supporting information

S1 File GA OMNI (September 2022). (DOCX)

S2 File Appendix A. (DOC)

S3 File SPSS data file. (SAV)

S4 File SPSS syntax. (SPS)

S5 File (DOC)
