# Supplementary material for: A theory-based assessment of mpox: Findings from a nationally representative survey of U.S. adults
Source: PLoS One. 2024 Mar 15;19(3):e0299599. doi: 10.1371/journal.pone.0299599 (PMC10942057; doi:10.1371/journal.pone.0299599)
Supplement: S1 File — (DOCX) [file pone.0299599.s001.docx]

**Section 1: Monkeypox**

Base: All respondents

MPX1 [S, GRID]

Please respond to each of the following statements with…

Statements in rows:

1. Monkeypox is present in the United States (correct)

2. You can get monkeypox from your infected pet (correct)

3. Monkeypox is always present in Western and Central Africa (correct)

4. There is a currently an outbreak of monkeypox in humans around the world (correct)

5. Monkeypox is caused by a virus (correct)

6. Human-to-human transmission of monkeypox occurs by prolonged exposure to an infected person’s skin lesions (correct)

7. Monkeypox and smallpox have similar symptoms (correct)

8. Skin lesions are one of the symptoms of monkeypox (correct)

9. Monkeypox has an average incubation period of 21 days (correct)

10. If you test positive for monkeypox you should isolate yourself for at least 2 to 4 weeks (correct)

11. Antibiotics are used to treat human monkeypox (incorrect)

12. Diarrhea is one of the symptoms of monkeypox (incorrect)

13. Vaccination is available to prevent monkeypox (correct)

14. Anti-viral medication is available to treat monkeypox (correct)

15. Only gay and bisexual men can get monkeypox (incorrect)

Answers in column:

1. No

2. Yes

3. I don’t know

Base: All respondents

MPX2 [S, GRID]

Please respond to each of the following statements with…

Statements in rows:

1. The U.S. government did not prevent the 9/11 attacks in order to justify the Afghanistan and Iraq campaigns.

2. A cure for cancer already exists, but the pharmaceutical industrial complex keeps it secret in order to maximize its profits.

3. The current financial crisis and recession represent the intended result of deliberate actions by banks, financial speculators and multinational corporations.

4. The financial crises of the last decade have been deliberately caused by political and financial authorities.

5. The U.S. government knew in advance about the 9/11 attacks, and deliberately decided not to stop them.

6. Soft-drink corporations add addictive substances in their products.

7. Infectious diseases are deliberately spread by governments and pharmaceutical multinationals.

8. The so-called Islamic State does not really exist; it is a smoke-screen concocted by Western Governmental Agencies.

9. LGBT groups have a plan to turn people into homosexuals by means of bogus “gender theories”.

10. Vaccines are useless and dangerous, they are only instrumental to the financial interests of pharmaceutical companies.

11. Groups of powerful people use chemtrails to poison the air, or to manipulate the environment.

12. Few groups of powerful individuals (George Soros, the Illuminati, the Rothschild family, the Free Masonry, etc.) secretly rule the world.

13. Monkeypox was bioengineered in a lab.

14. Monkeypox was intentionally released by scientists to deflect attention from the failures of the Biden administration.

15. Monkeypox is caused by exposure to a 5G signal.

Answers in column:

1. Strongly disagree

2. Disagree

3. Somewhat disagree

4. Neutral/no opinion

5. Somewhat agree

6. Agree

7. Strongly agree

Base: All respondents

MPX3 [S, GRID]

Please respond to each of the following statements with…

Statements in rows:

1. I am afraid of monkeypox.

2. It makes me uncomfortable to think about monkeypox.

3. My hands become clammy when I think about monkeypox.

4. I am afraid of losing my life because of monkeypox.

5. When seeing news stories about monkeypox, I become nervous or anxious.

6. I cannot sleep because I’m worrying about getting monkeypox.

7. My heart races or palpitates when I think about getting monkeypox.

8. I am at risk of getting monkeypox.

9. It is likely that I will get monkeypox.

10. Individuals in my household are at risk of getting monkeypox.

11. I feel knowledgeable about my risk of getting monkeypox.

12. I believe that monkeypox is a severe health problem.

13. If I get monkeypox, I will get sick.

14. If I get monkeypox, I will die.

15. If I get monkeypox, other members in my household will get sick.

Answers in column:

1. Strongly disagree

2. Disagree

3. Neither agree nor disagree

4. Agree

5. Strongly agree

Base: All respondents

MPX4A [S, GRID]

Please respond to each of the following statements with…

Statements in rows:

1. I have received the smallpox vaccination.
2. I have received the monkeypox vaccination.
3. I have received the COVID-19 vaccination.

Answers in column:

1. No

2. Yes

Base: MPX4A/1 or 2 = 2 (Did not receive Monkeypox or smallpox vaccine)

MPX4B [S, GRID]

Please respond to each of the following statements with…

Statements in rows:

1. How likely are you to get the smallpox vaccine in the next 6 months?

2. How likely are you to get the monkeypox vaccine in the next 6 months?

Answers in column:

1. Definitely not

2. Probably not

3. Unsure

4. Probably

5. Definitely

Base: All respondents

MPX5 [S, GRID]

To avoid monkeypox infection, I will:

Statements in rows:

1. Stop working out at the gym

2. Wear gloves when in public

3. Use bleach to wipe down surfaces wherever I am

4. Wear a mask when I am around other people

5. Stay celibate

6. Limit my social interactions

7. Refrain from taking public transportation

8. Get vaccinated against monkeypox

9. Stop performing oral sex

10. Stop receiving oral sex

11. Use a condom for every sexual encounter

12. Wear a mask during sexual activities

13. Limit the number of social events I attend

14. Stop sharing food with others

15. Only eat my meals at home

16. Take a semester off of school

17. Take leave from work

18. Only take online/remote courses

19. Work remotely

20. Remove my children from school/daycare

21. Homeschool my children

22. Wipe down my groceries with bleach

23. Wipe down my mail/packages with bleach

24. Use essential oils

25. Use an air purifier at my home

Answers in column:

1. No, I will not make this change

2. Already doing this

3. If the CDC recommends doing this (or not doing this)

4. If someone I know gets monkeypox

5. If I get monkeypox

6. Not applicable

Base: All respondents

MPX6 [S]

The most recent time you looked for information about health or medical topics, where did you go first?

1. Books

2. Brochures, pamphlets, etc.

3. Family

4. Friend/Co-worker

5. Doctor or health care provider

6. Library

7. Magazines

8. Newspapers

9. Telephone information number

10. Complementary, alternative, or unconventional practitioner

11. Television

12. General Internet websites (e.g., cnn.com)

13. Government websites (e.g., cdc.gov)

14. Health websites (e.g., WebMD.com)

15. Bing

16. Google

17. Twitter

18. Instagram

19. TikTok

20. Truth Social

21. Facebook/Meta

22. Parler

23. SnapChat

24. YouTube

25. Reddit

26. Gab

Base: if pppa1634 = Male

MPX7 [S]

Are you circumcised or uncircumcised?

1. Circumcised

2. Uncircumcised

3. Not sure
