# Supplementary material for: A theory-based assessment of mpox: Findings from a nationally representative survey of U.S. adults
Source: PLoS One. 2024 Mar 15;19(3):e0299599. doi: 10.1371/journal.pone.0299599 (PMC10942057; doi:10.1371/journal.pone.0299599)
Supplement: S2 File — (DOC) [file pone.0299599.s002.doc]

**Attachment A.** **Demographic Profile and Other Supplemental Data Provided for Each Project for KnowledgePanel Respondents**

| **Variable** | **Values** |
| --- | --- |
| Age | Actual age in years |
| Age, 7 categories | 1 = 18-24; 2 = 25-34; 3 = 35-44; 4 = 45-54; 5 = 55-64; 6 = 65-74; 7 = 75+ |
| Age, 4 categories | 1 = 18-29; 2 = 30-44; 3 = 45-59; 4 = 60+ |
| Current Employment Status | 1 = Working full-time |
|  | 2 = Working part-time |
|  | 3 = Not working |
| Education, 5 categories | 1 = No high school diploma or GED |
|  | 2 = High school graduate (high school diploma or the equivalent GED) |
|  | 3 = Some college or Associate degree |
|  | 4 = Bachelor’s degree |
|  | 5 = Master’s degree or higher |
| Education, 4 categories | 1 = No high school diploma or GED |
|  | 2 = High school graduate (high school diploma or the equivalent GED) |
|  | 3 = Some college or Associate degree |
|  | 4 = Bachelor’s degree or higher |
| Gender | 1 = Male |
|  | 2 = Female |
| Household Income | 1 = Less than $10,000 |
|  | 2 = $10,000 to $24,999 |
|  | 3 = $25,000 to $49,999 |
|  | 4 = $50,000 to $74,999 |
|  | 5 = $75,000 to $99,999 |
|  | 6 = $100,000 to $149,999 |
|  | 7 = $150,000 or more |
| Household Members Age 0 to 17 | Total number of household members in age group |
| Household Members Age 18 or Older | Total number of household members in age group |
| Household Size | Total number of members in household |
| Housing Type | 1 = One-family house detached from any other house |
|  | 2 = One-family condo or townhouse attached to other units |
|  | 3 = Building with 2 or more apartments |
|  | 4 = Other (mobile home, boat, RV, van, etc.) |
| Marital Status | 1 = Now married |
|  | 2 = Widowed |
|  | 3 = Divorced |
|  | 4 = Separated |
|  | 5 = Never married |
| MSA Status | 0 = Non-Metro |
|  | 1 = Metro (as defined by U.S. OMB Core-Based Statistical Area) |
| Ownership Status of Living Quarters | 1 = Owned or being bought by you or someone in your household |
|  | 2 = Rented for cash |
|  | 3 = Occupied without payment of rent |
| Race/Ethnicity | 1 = White, non-Hispanic |
|  | 2 = Black, non-Hispanic |
|  | 3 = Other, non-Hispanic |
|  | 4 = Hispanic |
|  | 5 = 2+ races, non-Hispanic |
| State | State of residence |
| U.S. Census Region 4 | 1 = Northeast |
|  | 2 = Midwest |
|  | 3 = South |
|  | 4 = West |
| U.S. Census Division 9 | 1 = New England |
|  | 2 = Mid-Atlantic |
|  | 3 = East-North Central |
|  | 4 = West-North Central |
|  | 5 = South Atlantic |
|  | 6 = East-South Central |
|  | 7 = West-South Central |
|  | 8 = Mountain |
|  | 9 = Pacific |

**Additional Data Provided for All Interviews**

**Variable Description**

Start Time Date/time respondent began taking survey

End Time Date/time respondent finished completing survey

Duration Length of time in minutes for self-administration of the instrument for a respondent
